# Supplementary material for: The presence of a G-quadruplex prone sequence upstream of a minimal promoter increases transcriptional activity in the yeast Saccharomyces cerevisiae
Source: Biosci Rep. 2023 Dec 19;43(12):BSR20231348. doi: 10.1042/BSR20231348 (PMC10730334; doi:10.1042/BSR20231348)
Supplement: Supplementary Figures S1-S4 and Table S1 [file BSR-2023-1348_supp.pdf]

The presence of a G4 prone sequence upstream of a minimal promoter increases transcriptional activity in the yeast *S. cerevisiae*

Supplementary Figures and legends

Libuše Kratochvilová, Matúš Vojsovič, Natália Valková, Lucie Šislerová, Zeinab El Rashed, Alberto Inga, Paola Monti, Václav Brázda[§]

§Corresponding author:

Václav Brázda

Institute of Biophysics of the Czech Academy of Sciences, Královopolská 135, 61265 Brno, Czech Republic

vaclav@ibp.cz

Oligonucleotides	$I_{(0)}$ Tris-HCl	$I_{(KCl)}$ Tris-HCl + 100mM KCl	$I_{(KCl)}/I_{(0)}$
**PUMA**	1.75 ± 0.23	1.39 ± 0.04	↓ 0.79
**KSHV**	16.93 ± 0,08	19.22 ± 0.38	↑ 1.14
**KSHV-1NO**	25.36 ± 0.12	31.76 ± 0.44	↑ 1.25
**KSHV-2NO**	9.73 ± 0.61	25.69 ± 0.76	↑ 2.64
**KSHV-Mut2.0**	19.87 ± 0.87	23.89 ± 1.40	↑ 1.20
**KSHV-Mut1.5**	15.24 ± 1.20	10.14 ± 0.15	↓ 0,67
**KSHV-3NO**	6.98 ± 0.92	10.58 ± 0.73	↑ 1.51

Table S1. Fluorescence intensity I/I_0 determined from oligonucleotides with the potential to form G4s. The average fluorescence intensity of three repetitions was related to the fluorescence intensity of the blank (ThT with the appropriate buffer). The $I_{(KCl)}/I_{(0)}$ fold indicates the fold decrease (↓) or increase (↑) of fluorescence emission of samples in buffer with the addition of K+ ions compared to samples in Tris-HCl without KCl.

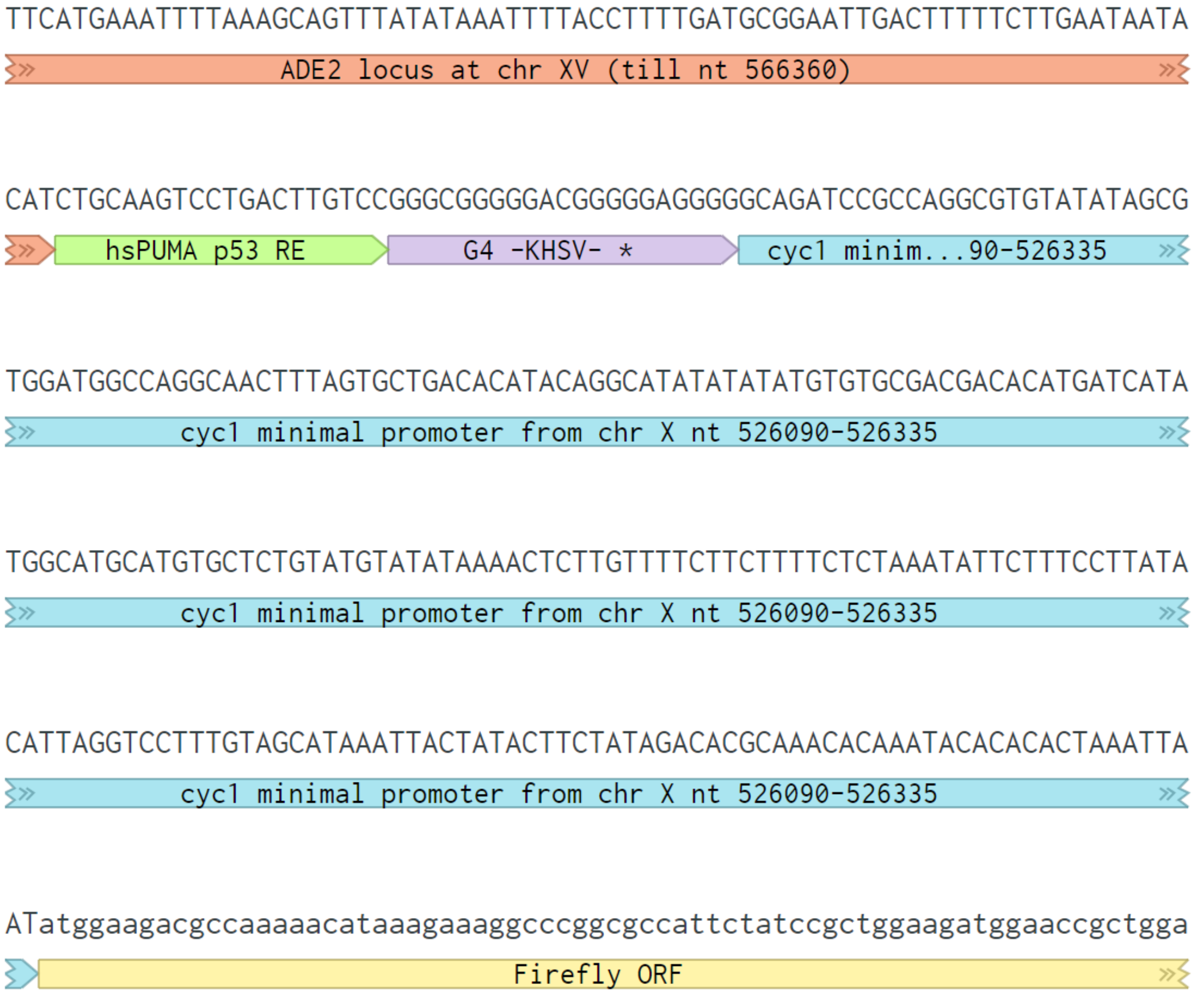

Figure S1. Sequence of the *ADE2* chromosomal locus edited to build the luciferase-based reporter assay. The *S. cerevisiae ADE2* locus on chromosomal XV was engineered as follows: first the *ADE2* open reading frame was replaced with the *Pothinus pyralis* firefly cDNA open reading frame (yellow annotation, the first ~70 nts are shown); concomitantly the *ADE2* promoter was replaced by a 245 nt portion of the promoter of the *CYC1* gene, corresponding to the sequence naturally present in chromosome X, from nucleotide 526090 till nucleotide 526335 (blue annotation) (Inga. 2022, ref 33). This sequence provides for low-level basal transcription of the luciferase gene. The locus has been further modified by placing upstream of the minimal CYC1 promoter the P53 RE derived from the human PUMA gene (hsPUMA p53 RE, green annotation) (Porubiakova, 2019, ref 25). Finally, for the experiments presented in this study, we constructed and used a panel of strains that differ only for the presence of a G4 prone sequence (purple annotation, the asterisk indicates that the KSHV is one of six elements that were tested, whose sequences are presented in Table 1) . The correct editing of the locus was confirmed by colony PCR and Sanger sequencing (see the Methods section for further details on the strain construction).

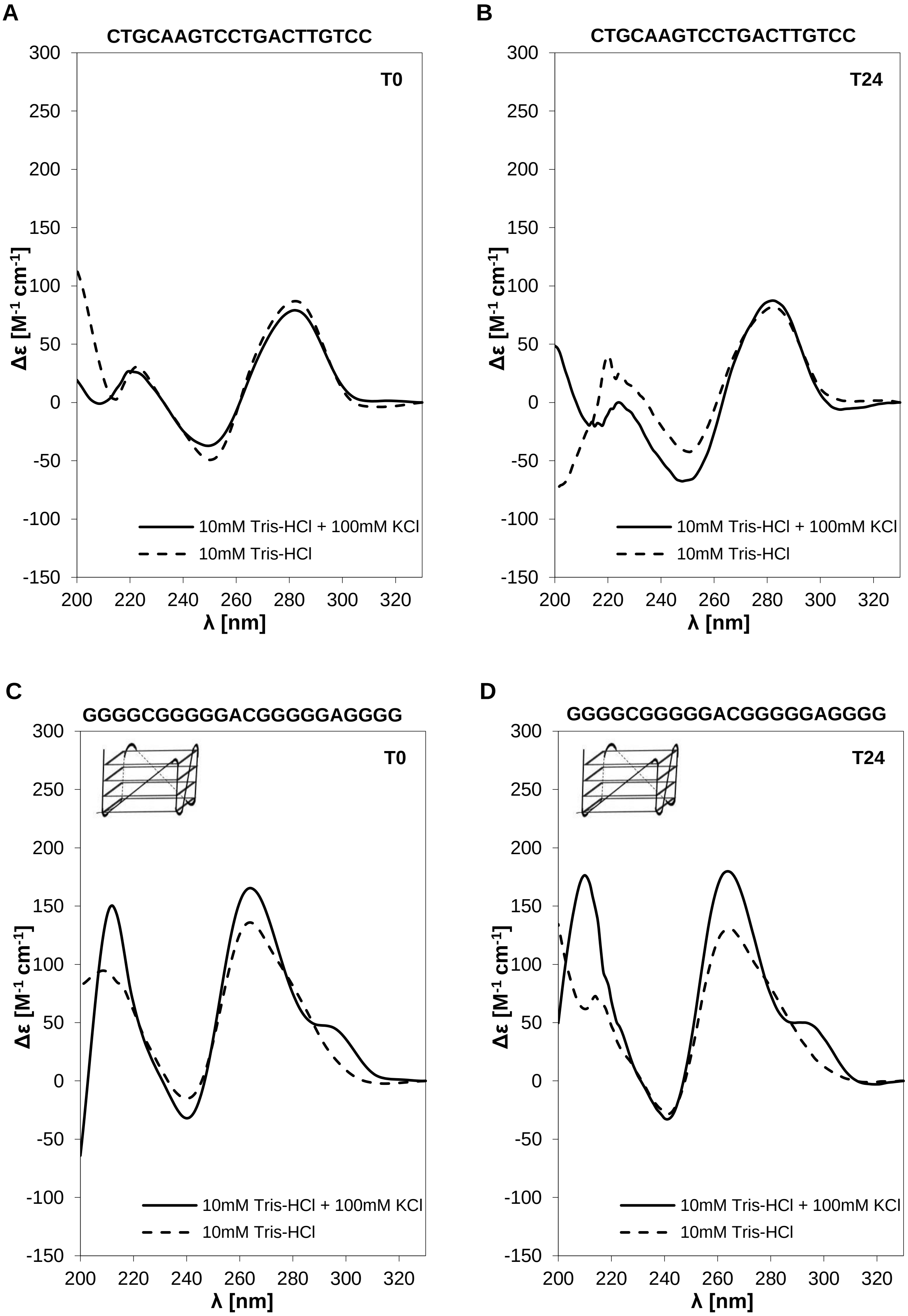
A
CTGCAAGTCCTGACTTGTCC
T0
B
CTGCAAGTCCTGACTTGTCC
T24
C
GGGGCGGGGGACGGGGGAGGGG
T0
D
GGGGCGGGGGACGGGGGAGGGG
T24
Δε [M-1 cm-1]
λ [nm]
300
250
200
150
100
50
0
-50
-100
-150
200
220
240
260
280
300
320
10mM Tris-HCl + 100mM KCl
10mM Tris-HCl

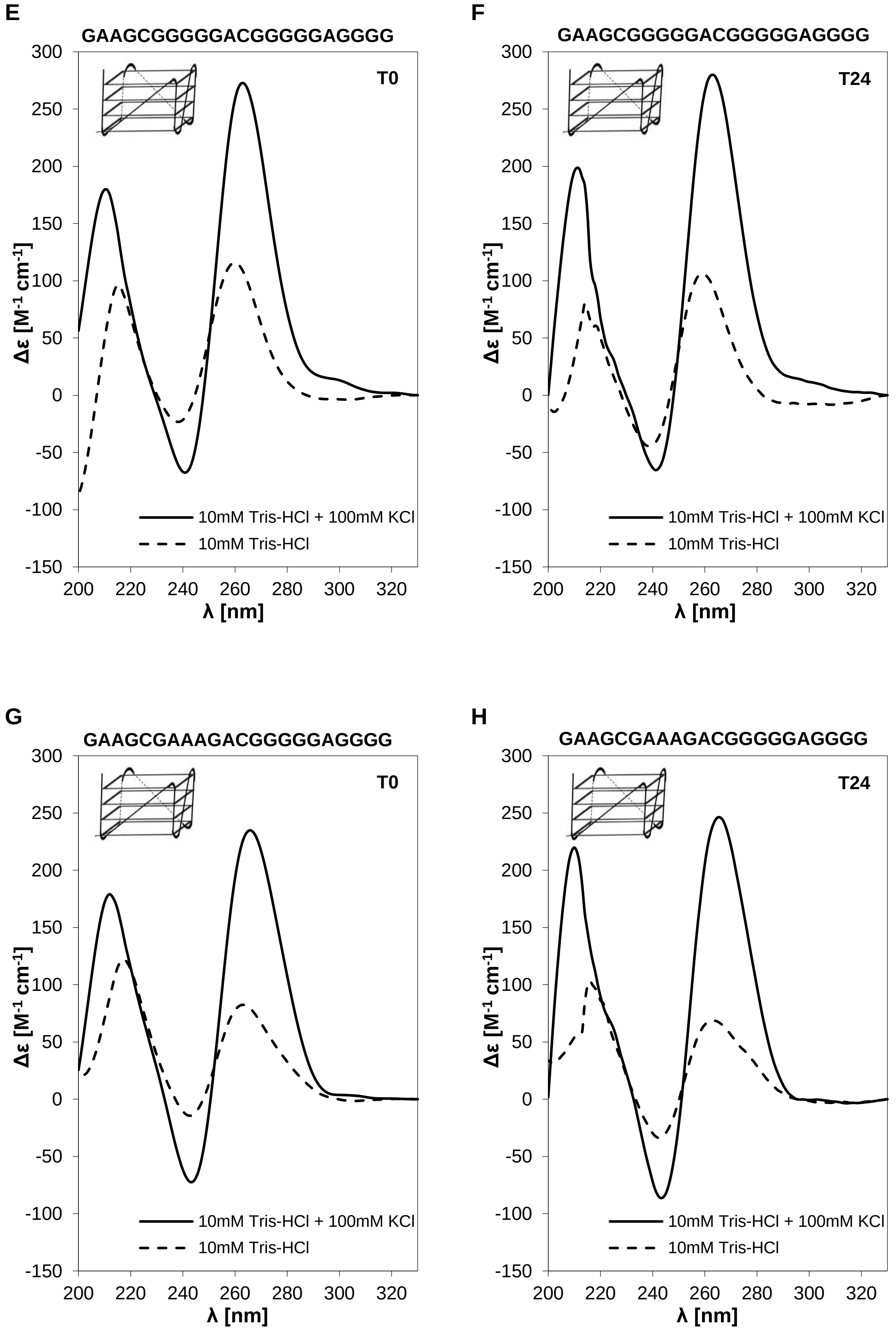
E
GAAGCGGGGGACGGGGGAGGGG
T0
F
GAAGCGGGGGACGGGGGAGGGG
T24
G
GAAGCGAAAGACGGGGGAGGGG
T0
H
GAAGCGAAAGACGGGGGAGGGG
T24
300
250
200
150
100
50
0
-50
-100
-150
Δε [M-1 cm-1]
200
220
240
260
280
300
320
λ [nm]
10mM Tris-HCl + 100mM KCl
10mM Tris-HCl

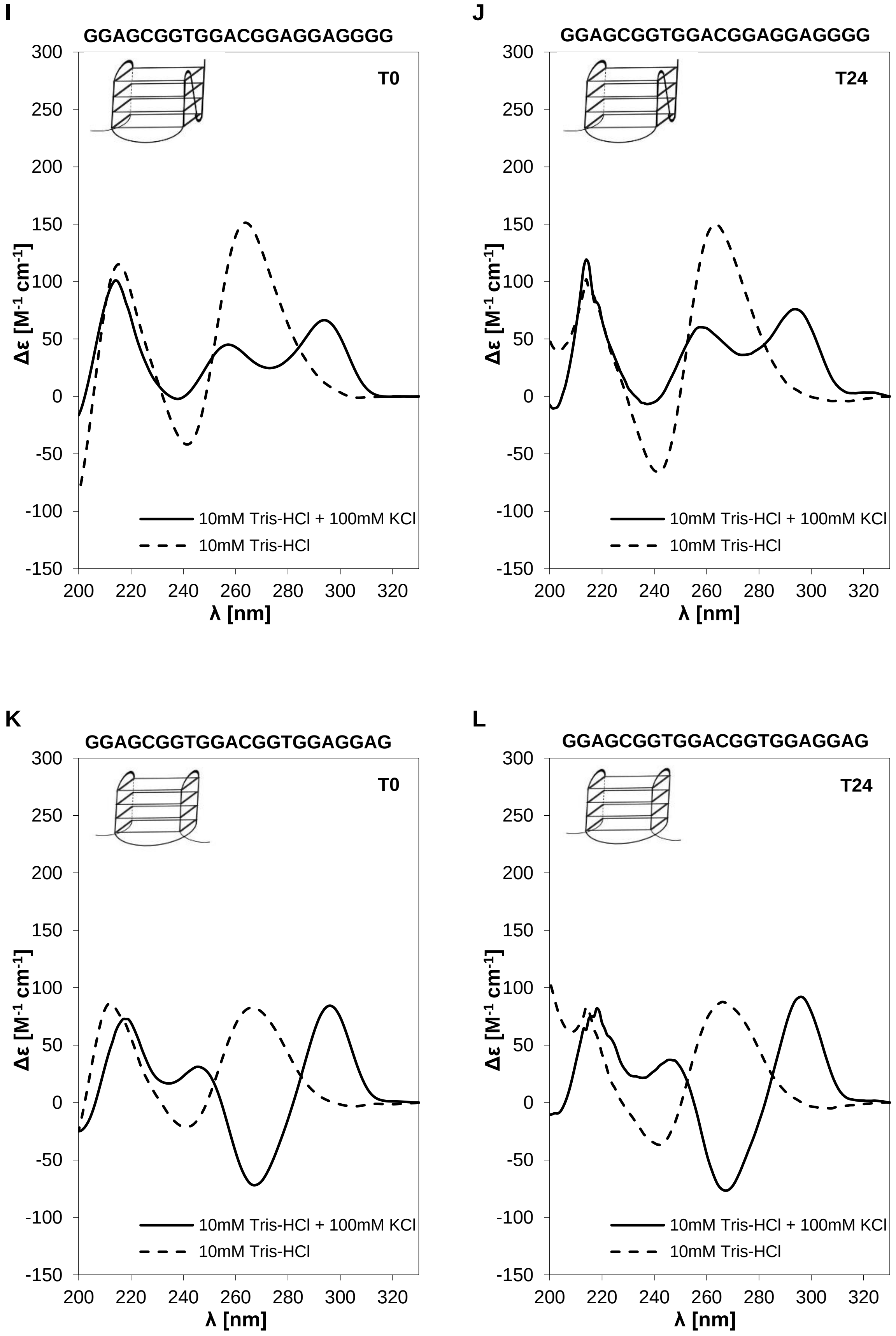
I
GGAGCGGTGGACGGAGGAGGGG
T0
J
GGAGCGGTGGACGGAGGAGGGG
T24
K
GGAGCGGTGGACGGTGGAGGAG
T0
L
GGAGCGGTGGACGGTGGAGGAG
T24
Δε [M-1 cm-1]
λ [nm]
10mM Tris-HCl + 100mM KCl
10mM Tris-HCl
300
250
200
150
100
50
0
-50
-100
-150
200
220
240
260
280
300
320

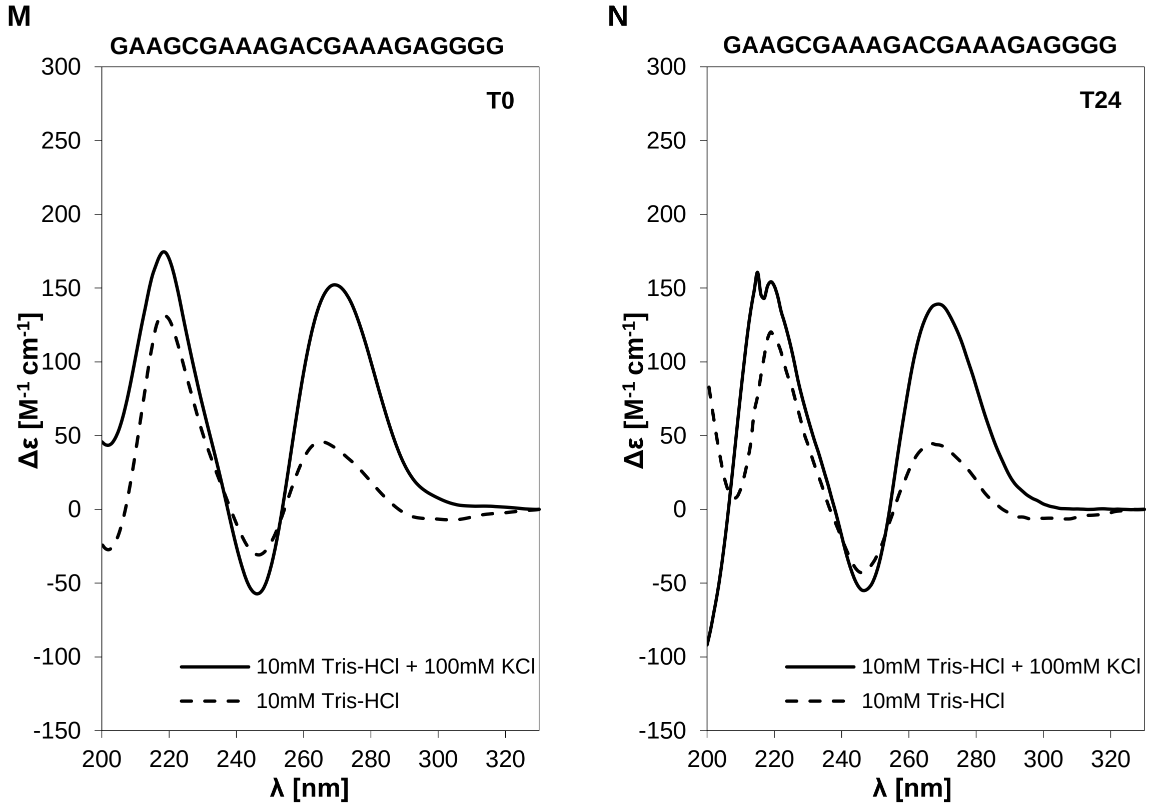

Figure S2. CD spectra of oligonucleotides under study at T_0 (left panel) and T_{24} (right panel). CD spectra of PUMA oligonucleotide **(A, B)**, KSHV oligonucleotide **(C, D)**, KSHV-1NO oligonucleotide **(E, F)**, KSHV-2NO oligonucleotide **(G, H)**, KSHV-Mut2.0 oligonucleotide **(I, J)**, KSHV-Mut1.5 oligonucleotide **(K, L)** and KSHV-3NO oligonucleotide **(M, N)**. The solid line shows the spectra of the sample hybridized in 10 mM Tris-HCl with the addition of 100 mM KCl. The spectra of the sample hybridized in the medium without the addition of K^+ ions are plotted as a dashed line. The CD spectra at T0 from Figure 2 were also reproduced here to facilitate comparisons between the two time points.

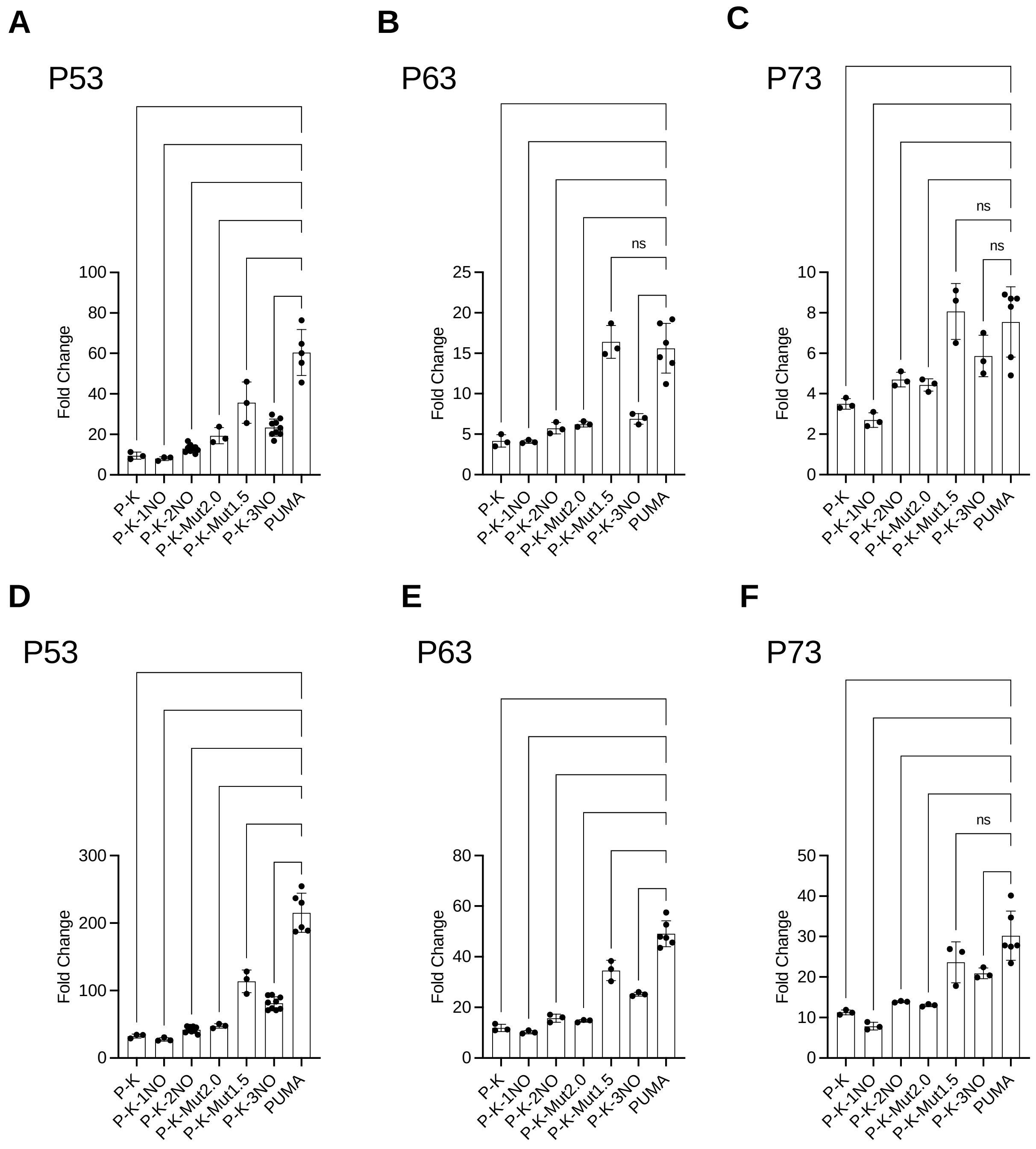

Figure S3. Effect of G4 prone sequences on P53 family transactivation. Fold change measurements for the indicated panel of yLFM reporter strains from P53, P63 and P73 yeast transformants at 0.016% galactose for 6 hours **(A-C)** or at 1% galactose for 6 hours (**D-F**)**.** Data are presented as mean ± standard deviation (SD) of at least three biological replicates. Individual values are also plotted. The symbols *, **, *** and **** indicate significant differences for p≤*0.0146*, p=0.0065, p=0.0006 and p<0.0001, respectively between PUMA strain and those containing other G4 regulatory elements. ns, not significant. Ordinary one-way ANOVA test.

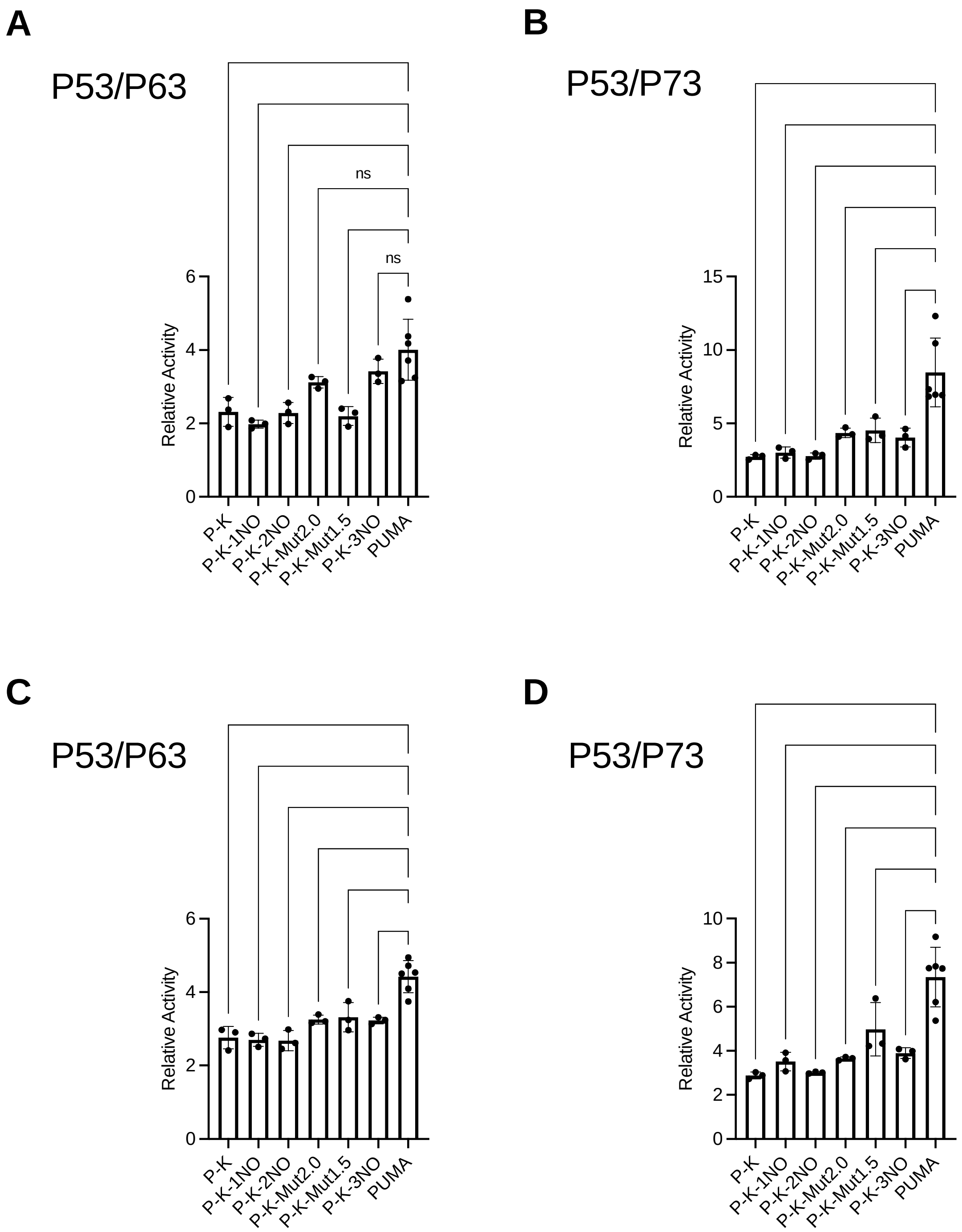

Figure S4. Effect of G4 forming sequences on P53/P63 and P53/P73 relative activity plotted as bars graph. (A), **(C)** P53/P63 relative activity at 0.016% and 1% Galactose, respectively in the indicated panel of yLFM reporter strains. **(B)**, **(D)** P53/P73 relative activity at 0.016% and 1% Galactose, respectively as above. Data are presented as mean ± standard deviation (SD) of at least three biological replicates. Individual values are also plotted. The symbols **, *** and **** indicate significant differences for $p \leq 0.0064$, $p=0.0009$, and $p<0.0001$, respectively between PUMA strain and those containing other G4 regulatory elements. ns, not significant. Ordinary one-way ANOVA test.
